# Editorial: “A Touch of Physiotherapy”—The significance and meaning of touch in the practice of physiotherapy

**DOI:** 10.3389/fresc.2023.1161574

**Published:** 2023-04-06

**Authors:** W. S. Bjorbækmo, T. Dahl-Michelsen, D. A. Nicholls

**Affiliations:** ^1^ Department of Rehabilitation Sciences and Health Technology, OsloMet - Oslo Metropolitan University, Oslo, Norway; ^2^ Department for Interdisciplinary Health Sciences, University of Oslo, Oslo, Norway; ^3^ Department of Health, VID – Specialized University, Oslo, Norway; ^4^ School of Clinical Sciences, Auckland University of Technology, Auckland, New Zealand

**Keywords:** touch, health care, haptic, therapy, history, future practice

**Editorial on the Research Topic** “A Touch of Physiotherapy”—The significance and meaning of touch in the practice of physiotherapy

Touch is a vastly underexamined concept in physiotherapy which for years has almost exclusively focused on technical application related to pathologies (1–8). Nowadays, haptic studies are experiencing a revival as people experience touch as a principal factor for the spread of viral disease, and digital technologies have put many of our interactions beyond arm's-length (9, 10).

People are craving authentic, intimate, and skilled touch like never before and through touch seek all manner of ways to reconnect with their bodies, others, and the world (7, 11). At the same time, healthcare services around the developed world are struggling to cope with escalating healthcare needs and are, in many places, responding by increasing productivity and throughput at the expense of personalised care (12). Some of this has been made necessary by the pandemic and the ease with which we now can connect with each other through computer screens (13). So, just as people are craving touch in their personal lives, healthcare systems all around the world are abandoning this (14).

In physiotherapy, this turn away from touch has been accelerated in recent years by a growing evidence-based practice movement arguing that touch equates to “low value care”, meaning care that is expensive to deliver and offers questionable efficacy (15–17). Many physiotherapists now argue that practitioners should not be touch-based, but instead concentrate on greater personal responsibility, activity-based therapies, and cognitive/hands-off forms of personal re-education (18).

In many ways, the emergence of psychologically informed therapies including cognitive functional therapy, acceptance and commitment therapy, and exercise-based rehabilitation for long-term chronic health problems, represent a tacit criticism of the manual therapies that were once the cornerstone of physiotherapy practice (19, 20).

This is not the first time we have seen touch-based physiotherapy go out of fashion. We saw it after World War I, when individual hands-on care was replaced by exercise-based approaches as efficient ways to rehabilitate large numbers of people (21). A similar decline occurred in the 1960s with the widespread uptake of electrotherapy devices to deliver physiotherapy in an efficient way to large numbers of people (18).

In both cases the decline of popularity of touch coincided with periods when healthcare economics drove practitioners towards ways of administering physiotherapy to the largest possible number of people at the lowest cost (18). We can see this happening again today with widespread concerns about the rising cost of publicly funded health care, the constant strain being felt by public health services, and a 50-year project to open the protective enclosures surrounding public health services to market competition and neoliberal health-care reforms. It is perhaps no surprise then that many of the modalities that are becoming popular in physiotherapy today explicitly encourage individuals to take more personal responsibility for their own health (22, 23).

Given this, it is unlikely that touch will return to being a central focus of physiotherapy professional practice for some time. Economic and political conditions will need to change so that people, once again, demand, and have supported access to, forms of touch-based therapy (13, 14, 24, 25).

For many physiotherapists, the lack of touch in their practice is the cause of great existential distress because touch is one of the cardinal features of their professional identity (1, 26). It is one of the main ways in which they distinguish their practice from the practice of others. After all, a person can get perfectly adequate exercise advice and cognitive behavioural support from any number of online sources. But you cannot get skilled, caring, empathic touch from a robot or a Facebook feed.

What this collection of seven papers highlight is that touch remains a very vibrant and important subject in physiotherapy, and that perhaps for the first time it is starting to receive serious critical scrutiny. The papers represent some of the breaths and diversity of ideas starting to emerge around touch in the 21st-century. They are, of course, only tasters and so much more work is needed. But they do make a strong claim that touch remains perhaps the sentinel modality distinguishing physiotherapy from every other discipline and, not (4, 5) withstanding its many critics, these authors see a bright future for touch as an important physical therapy for the planet.

Based on embodied enactive perspective, Sørvoll et al. argue that contrary to the idea of touch as a passive approach to therapy, touch intertwines social and environmental concerns that are essential if we are to engage and facilitate children's movement in physiotherapy.

Focusing on therapeutic handling, Håkstad et al. argue that we need to move beyond the dichotomizing debate of hand-on vs. hands-off approaches in pediatric physical therapy.

Bjorbækmo and Mengshoel highlight how multiple dimensions of touch are dynamically created, developed, and expressed in situations where the physiotherapeutic treatment goal might be known, but the path (the physiotherapist know-how) to achieving it remains open and uncertain.

Through three empirical examples anchoring physiotherapy practice in an extended framework based on phenomenology, social sciences, and new knowledge from neurosciences, Thornquist challenges the proposition that hands-on clinical work turns patients into “passive” recipients.

Using a single physiotherapy case theoretically framed within theories about the meta functions of language, Ahlsen and Nilsen illuminate how verbal and nonverbal communication are used by physical therapists—showing how interruptions, repetitions, unfinished sentences, touch, and gaze facilitate patient's participation in the physiotherapy encounter.

Drawing from cases in neurological and musculoskeletal practice, Tuttle and Hiller suggest that the false dichotomy between task-specific training and touch, and active movements and massage should be addressed in the learning and teaching of physiotherapy students.

And finally, Nicholls explores how touch in physiotherapy is narrowly humanistic, both as a bio-physical and inter-subjective phenomenon. Drawing on Deleuze's machine ontology he offers another point of view which contributes a radically pluralistic new thinking enabling physiotherapists to move beyond current practice understanding of touch.

Each paper highlights nuanced understandings of the phenomenon of touch. To fully understand the possibilities for touch, not only as a human relational modality but also as a fundamental ontological feature of all forms of life throughout the universe, this special issue argues that touch may be one of the defining concepts underpinning physical therapy practice.
